# K-clique percolation in free association networks and the possible mechanism behind the $7 \pm 2$ law

**DOI:** 10.1038/s41598-022-09499-w

**Published:** 2022-04-01

**Authors:** Olga Valba, Alexander Gorsky

**Affiliations:** 1 Department of Applied Mathematics, MIEM, National Research University Higher School of Economics, Moscow, 123458 Russia; 2 Kharkevich Institute for Information Transmission Problems RAS, Moscow, 127051 Russia; 3 Moscow Institute of Physics and Technology, Dolgoprudny, 141700 Russia

**Keywords:** Neuroscience, Mathematics and computing

## Abstract

It is important to reveal the mechanisms of propagation in different cognitive networks. In this study, we discuss the k-clique percolation phenomenon as related to the free association networks including the English Small World of Words project (SWOW-EN). We compared different semantic networks and networks of free associations for various languages. Surprisingly, k-clique percolation for all $k<k_c=$ 6–7 is possible on free association networks of different languages. Our analysis suggests new universality patterns for a community organization of free association networks. We conjecture that our result can provide a qualitative explanation of Miller’s $7\pm 2$ rule for the capacity limit of working memory. A new model of network evolution extending the preferential attachment is suggested, providing the observed value of $k_c$.

## Introduction

Networks represent powerful models for exploring different cognitive systems and processes^1,2^. For example, in^3,4^, the authors propose a multiplex network model of the formation of mental lexicon and early word acquisition. In^5^, the author discusses the structural properties of semantic networks for low and high creativity people. In^6–10^, network-based methods are used to simulate the mechanisms of solving Remote Associates Tests, allowing one to estimate a human’s creative potential^11^.

Complex networks often exhibit meso-scale or global characteristics of structural order. Certain networks exhibit community structure, in which densely connected communities of nodes exhibit sparse or weak inter-community connections. In semantic networks, one word can belong to several communities, so standard community detection methods are not applicable. We investigate the community organization of the free association network, focusing on one described in^12^, known as English Small World of Words project (SWOW-EN). This network differs from other datasets in its higher density, which is achieved by the presence of links of weak association strength. The dense network structure allows us to study a k-clique community organization of larger k. We compared its properties with various semantic networks of the English and Dutch languages and networks of free associations.

This study is mainly focused on percolation analysis of the free association networks. The percolation approach was used to quantify the flexibility of one or another network characteristics of semantic network^13–15^. In^13^, flexibility of thought was investigated by percolation analysis and the cognitive declines due to aging are discussed. In context of the creativity theory the percolation analysis has been discussed in^5,13,14^, demonstrating that the semantic network of the high-creative group broke apart slower than that of less-creative group. It was also shown via a percolation approach in^14^ that the mental lexicon is fragile against progressive word failure with multiplex network attacks across the lifespan.

A more general phenomenon involves the percolation of k-clique introduced in^16,17^. For the random Erdos-Renyi ensemble, the critical link probability for any k can be found analytically. However, for real networks, an estimation of the critical threshold for k-clique percolation is a nontrivial problem. In cognitive networks, the k-clique percolation has been recently discussed in^18^ related to the problem of aging in the semantic memory.

In this study, we investigate a k-clique percolation in the free association networks and question if there is some upper boundary $k_c$ where no k-clique percolation exists for $k>k_c$. A bit surprisingly, there is a sharp boundary at $k_c=$ 6–7 for the English and Dutch languages.

The sharp boundary for clique percolation certainly provides information concerning the structural organization of free association networks. However, it also influences the effectiveness of the processes on the network, since k-clique percolation is a particular dynamical process. Only the k-cliques with $k<k_c$ can propagate effectively through the free association network. A discussion concerning the distinction between a structure and a process in semantic networks can be found in^19,20^.

The test protocols for free associations allow very short time intervals for answering; hence, we can consider them a kind of probe of working memory. On the other hand, the limitation of the working memory capacity is a well-known phenomenon^21–23^. A person can remember only a finite number of items of different nature simultaneously, although there is some mild dependence on the nature of an item. This phenomenon is known as Miller’s $7\pm 2$ law. We conjecture that our finding could serve as a potential explanation of the mechanism behind Miller’s law. We have to remember the k-linked items for some short period of time; this can be considered as the k-clique percolation process in some effective “working memory network”.

Looking at the mechanism responsible for the limit of working memory capacity, the natural question concerns the evolutionary origin of the particular value of $k_c$ and the rules of evolution that bring the network to this particular value of $k_c$. We suggest a new rule of network evolution which can be considered as the modification of preferential attachment when a new node is linked to two others connected to each other. This new mechanism provides the desired value $k_c=$ 5–6 for various sizes of the network.

## Methods

### Data description

The free association network SWOW-EN is a weighted directed network with $N=$ 12 217 stimuli words. Stimulus materials (cue words) were constructed using a snowball sampling method, allowing authors^12^ to include both frequent and less frequent cues at the same time. The final set consists of 12 292 cues (stimuli); the weight of the link indicates the fraction of the experiment participants who gave this particular response to a cue and can be considered as the conditional probability of a response given a cue. Therefore, the total weight of links going out of each node is lower than or equal to 1. For our analysis, we considered the network as undirected, attributing the greatest weight to an edge in the case of a bidirectional association.

We also analyzed the free association network, based on the South Florida Free Association data base^24^ and the free association network, known as the Edinburgh Associative Thesaurus^25^.

We used networks, containing various types of relations. The phonological network captures the phonological similarities, which are based on IPA transcription from WordNet 3.0^27^. Words A and B are connected if they have IPA transcriptions with an edit distance of one. The network of synonyms contains the coupled words with the same meaning. Many words refer to categories that are taxonomically organized, e.g.,“horse”is a type of “animal”. This taxonomic organization results into basic, superordinate and subordinate-level object categories.The taxonomic network contains hyponymy relationships between words. All data were retrieved from Wolfram Research^26^, which mostly coincides with WordNet 3.0^27^. The multiplex network contains all these three types of word relations.

Finally, we studied free association networks for the Russian and Dutch languages. We used Russian thesaurus^28^ and Dutch association data^29^, removing words, that have no associations. Table  1 summarizes the basic structural properties of used networks.

**Table 1:** Structural properties of semantic networks.

Network	Nodes	Edges	Density	Transitivity	Clustering	$p_c(2)$	$p_c(3)$
SWOW-EN free association	12 217	352 403	0.0047	0.052	0.113	$8.2\cdot 10^{-5}$	0.0064
Florida free association	5 019	55 246	0.0044	0.083	0.186	$2.0\cdot 10^{-4}$	0.0100
Edinburgh free association	8 210	241 461	0.0072	0.048	0.103	$1.2\cdot 10^{-4}$	0.0078
Taxonomic	7 943	42 042	0.0013	0.048	0.093	$1.3\cdot 10^{-4}$	0.0079
Synonyms	6 526	13 134	0.0006	0.284	0.344	$1.5\cdot 10^{-4}$	0.0088
Phonological	4 618	15 447	0.0014	0.345	0.246	$2.2\cdot 10^{-4}$	0.0104
Multiplex	8 383	68 505	0.0019	0.112	0.283	$1.2\cdot 10^{-4}$	0.0078
RUS thesaurus	5 377	51 191	0.002	0.067	0.163	$1.9\cdot 10^{-4}$	0.0096
Dutch data	10 486	207 810	0.0038	0.067	0.163	$9.5\cdot 10^{-5}$	0.0069

### K-clique percolation

We begin with a few definitions, laying down the fundamentals of k-clique percolation^16,17^. *K-clique* is a complete (fully connected) subgraph of k vertices. We say, that two k-cliques are *adjacent* if they share $k-1$ vertices, i.e., if they differ only in a single vertex. A subgraph, which is the union of a sequence of adjacent k-cliques, is called *k-clique chain*, and two k-cliques are *k-clique-connected*, if there exists at least one k-clique chain containing the two k-cliques. Finally, *k-clique percolation cluster* is defined as a maximal k-clique-connected subgraph, i.e., it is the union of all k-cliques that are k-clique-connected to a particular k-clique.

The Erdosh–Renyi random graphs show a series of interesting transitions when the probability *p* of two nodes being connected is increased. For $k=2$, the transition is well known and manifested by the appearance of a giant component in a network at critical probability $p_c(k=2)=\frac{1}{N}$, where *N* is the number of nodes. For each *k*, one can find a certain threshold probability $p_c(k)$ above which the k-cliques organize into a giant community^17^:

$$\begin{aligned} p_c(k)=\frac{1}{\left[ N(k-1)\right] ^{\frac{1}{k-1}}}. \end{aligned}$$

Table  1 contains the values $p_c(k)$ with $k=2,3$ for random networks of the same size as semantic networks. We found, that network density,i.e., the observed link probability, for all datasets satisfies the inequality $p_c(2)<\rho <p_c(3)$. That is, if links in a semantic network were formed randomly, then all the vertices are included in the percolation cluster of $k = 2$, that is,one connected component, but do not form a cluster of $k = 3$.

## Results

### K-clique community organization of semantic networks

**Figure 1:**
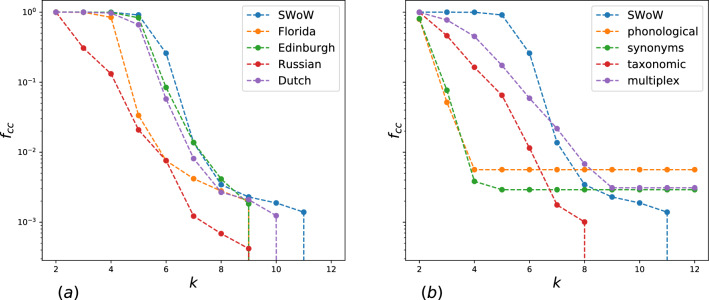
(**a**) The size of k-clique percolation cluster in dependence on the value k for different free association datasets. (**b**) The size of k-clique percolation cluster in dependence on the value k for different English semantic networks.

We calculated the fraction of nodes $f_{cc}$, included in k-clique percolation cluster for different values k. The dependencies for different free association datasets are presented in Fig. 1a. Firstly, note that almost all words are included in a 3-clique percolation cluster, the existence of this cluster explains the high transitivity and average clustering coefficient, see Table  1. Secondarily, all free association networks demonstrate k-clique percolation for large k, i.e., the clusters of $k=5$ and $k=6$ contain essential fractions of words—and, for in the SWoW-EN dataset, almost all words. Of course, analyzing free association data, it is necessary to consider the various data collection conditions, which are primarily related to properties such as the network size and its density. These properties completely determine the k-clique percolation clusters for random graphs^17^. We believe that k-clique organization of free association networks is of universal nature and does not essentially dependence on the network size and density. This universality allows us to propose a new mechanism for the growth of the semantic networks. The network density increases with weak associations included, so we discuss k-clique percolation depending on the association strength in details. We analysed the robustness of k-clique organization of free association networks, simulating two mechanisms of node removing. In the first scenario, we choose randomly $\alpha N$ nodes. In the second model, we took $\alpha N$ nodes with the smallest degree. The respective k-clique percolation dependencies for different datasets are presented in Supplementary Information Fig. S1). We observe the natural changes of k-clique percolation with increasing of the numbers of nodes removed randomly, while k-clique organization in the second process are stable. This indicates to the universality of the k-clique organization of free association networks.

In Fig. 1b, the dependencies for semantic networks of different natures are presented. In contrast to the networks of free associations, phonological and synonymous networks form a 3-clique percolation clusters only partially; clusters of higher orders are completely absent, despite the fact that these networks are characterized by higher values of transitivity and clustering. We also calculated the respective dependence for the so-called multiplex network, in which we considered three layers:phonological, taxonomic and synonyms. For such a network, we observe clusters of the order of 4 and 5. Thus, we can assume that the variety of links ensure the existence of high-order clique clusters.

### Structural features and clustering in the SWOW-EN network

**Figure 2:**
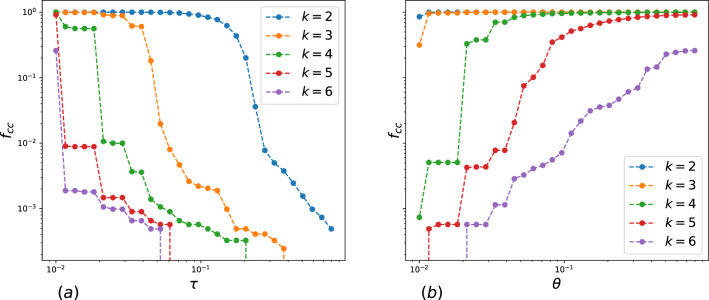
(**a**) The size of k-clique percolation cluster in dependence on the threshold $\tau$ for different values k in SWOW-EN. (**b**) The size of k-clique percolation cluster in dependence on the threshold $\theta$ for different values k in SWOW-EN.

**Figure 3:**
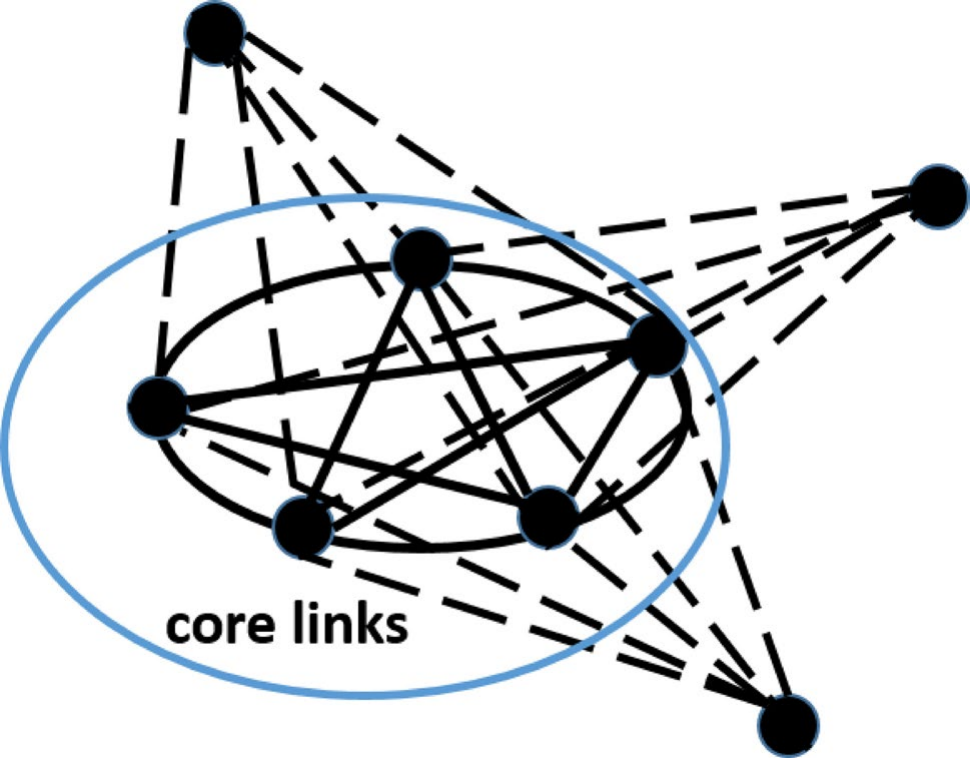
(**a**) Three k-cliques are adjacent ($k=6$) through the central (k − 1)-clique, which could be considered as a“core”.

We analyzed k-clique community clusters depending on the association strength. For this aim, we performed the following numerical experiments. In the first simulation, we took a threshold $\tau$ for association strength and deleted all links of weights *less* than the threshold. Figure 2a presents the fraction of nodes including in k-clique community cluster of $k=2,3\ldots 6$ depending on the threshold $\tau$. We observe, that k-clique community clusters of higher order ($k=5$ and $k=6$) exist only for initial network state and almost disappear at a small threshold. Percolation clusters for $k = 3$ and $k=4$ include all vertices up to sufficiently high threshold $\tau$, indicating the stability of network community organization. The second simulation was as follows. We established a threshold $\theta$ for association strength and deleted all links of weights *more or equal* than the value $\theta$, i.e. we analyzed a subgraph of weak associations. The respective dependencies for different k are depicted in Fig. 2b; the 3-clique percolation cluster is not sensitive to the threshold $\theta$ and exists for all weak subgraphs. The percolation clusters for $k = 4$ and $k=5$ include all words for high threshold and abruptly decrease at small values $\theta$. Interestingly, that for $k=6$ (even for very high $\theta$), the percolation cluster contains only some parts of nodes. This result shows that strong free associations can be considered as“core”links, which are involved in few cliques, providing intersections of cliques in percolation clusters, while weak associations form rather a“shell”of clique community - see Fig. 3.

**Figure 4:**
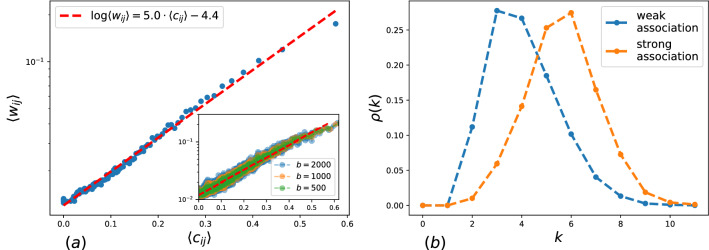
(**a**) The dependence of average association strengths on the edge clustering. Insert: the dependencies for different number of bins *b*. (**b**) The distribution of maximal clique sizes for weak ($w_{ij}\le 0.01$) and strong ($w_{ij} >0.1$) associations.

This assumption is confirmed by studying the distribution of triangles belonging to the links depending on association strength. We introduce an *edge clustering coefficient* for as follows:

$$\begin{aligned} C_{ij}=\frac{N_T(ij)}{\min (k_i, k_j)-1}, \end{aligned}$$

where $N_T(ij)$ is the number of triangles, containing the edge (*i*, *j*), $k_i, k_j$ are the degrees of *i* and *j* nodes respectively. Like the clustering coefficient of a node, the value $C_{ij}$ shows the fraction of triangles and lies in the range $\left[ 0,1\right]$. Note that the introduced clustering coefficient $C_{ij}$ correlates with a topological overlap for nodes *i* and *j* in case of their adjacency^30^. We found the clustering coefficient for each edge in the free association network SWOW-EN, sorted them, and splitted them into $b = 100$ intervals of equal size. For each intervals $l, l=1,2,\dots ,n$, we calculated the average values for the clustering coefficient $\left<c_{ij}^l\right>$ and for the association strengths $\left<w_{ij}^l\right>$, which are equal to the fraction of the experiment participants who gave this particular response to a cue. Figure 3a presents the dependence of the average association strength on the respective clustering coefficient in given interval. The dependence is fitted by the curve $\log \left<w_{ij}\right>=5.0\cdot \left<c_{ij}\right>-4.4$. Thus, we observe a positive correlation between the number of triangles, belonging to a link and its association strength. Note that this correlation has not been discussed before and it is interesting by itself and can be used in modeling the human lexicon. Besides, we introduce *k-clique number* of an edge as the maximal clique size, containing the edge. In Fig. 3b, the distributions of k-clique numbers are presented for the weakest association links, i.e. $w_{ij}=0.01$ and for the strongest association links, $w_{ij}>0.1$, the fractions of the weakest and the strongest links are $20\%$ and $5 \%$ respectively.

### Simulation of clique organization in free association networks

Network models of language structure are discussed in^31,32^. Particularly, Dorogovtsev and Mendes^32^ proposed a stochastic theory of the evolution of human language, which treats language as a self-organizing network of interacting words. It is well known that language evolves. Thus, the question is what kind of growth (in the sense of increase of lexical repertoire) leads to a self-organized structure with characteristic scale-free degree distribution. Dorogovtsev and Mendes’scheme of the language network growth follows. A new word is connected to an old one *i*, with the probability proportional to its degree $k_i$ (Barabasi and Albert’s preferential attachment); additionally, at each time step, *c*, new edges randomly emerge between old words, where *c* is a constant coefficient that characterizes a particular network. This model explained power law degree distribution and small-world properties of semantic networks very well (Fig. 4).

**Figure 5:**
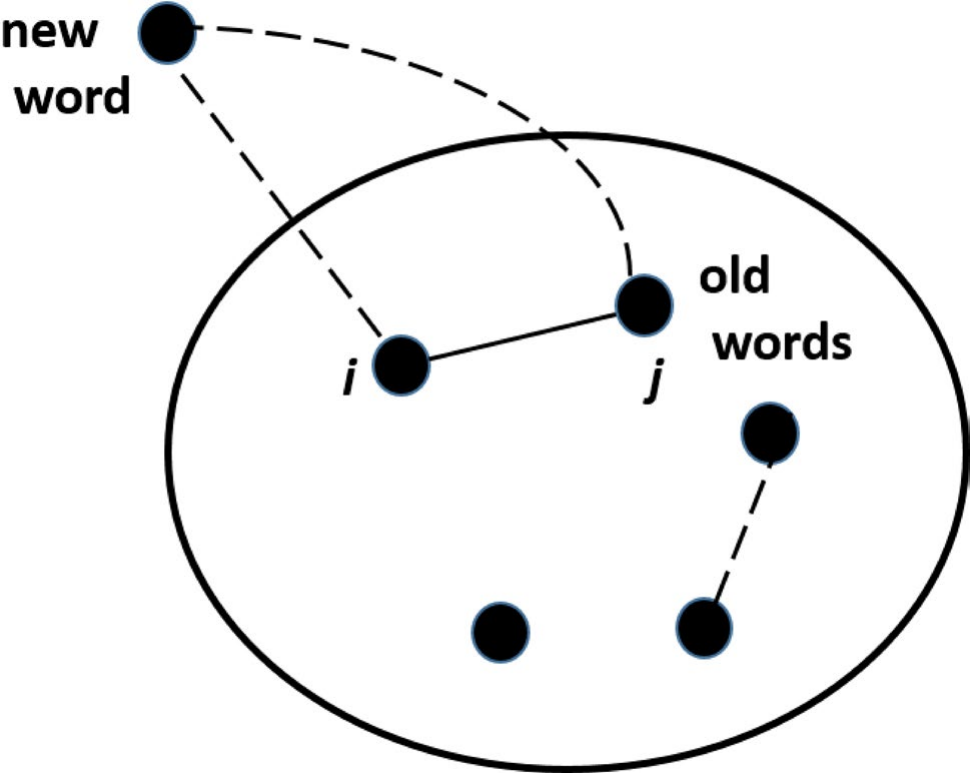
Network model description: a new word is connected to a link (*i*, *j*) by preferential attachment; in addition, random links between old words emerge. Existing links are depicted by solid line, new links are dashed.

To describe clique organization in semantic networks we propose a new model based on Dorogovtsev and Mendes’mechanism, presented in Fig. 5a. In our model a new word is connected to 2*m* existing *linked* words *i* and *j* with the probability proportional to the sum degree $k_i+k_j$, forming a triangle; in addition, at each time step, we add *c* new edges randomly between old words. The network evolution begins with an initially small Erdos-Renyi random graph $G(l,p_0)$.

**Table 2:** Structural properties of simulated networks.

Nodes	Edges	Density	Transitivity	Clustering	$p_c(2)$	$p_c(3)$
2000	23 213	0.0116	0.048	0.175	$5\cdot 10^{-4}$	0.0158
4000	46 783	0.0058	0.028	0.158	$2.5\cdot 10^{-4}$	0.0111
6000	69 307	0.0039	0.016	0.172	$1.66\cdot 10^{-4}$	0.0091
8000	91 275	0.0028	0.010	0.187	$1.25\cdot 10^{-4}$	0.0079

**Figure 6:**
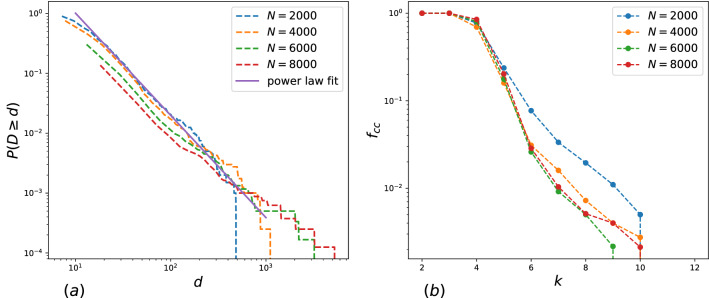
(**a**) Complementary cumulative degree distribution function for simulated networks of different sizes. (**b**) The size of k-clique percolation cluster in dependence on the value *k* for networks of different sizes.

We simulated the networks of different sizes with the model parameters $m=4$, $c=4, l=20, p_0=0.2$. Structural properties of the networks are summarized in Table  2. All networks are sparse, with density, $p_c(2)<\rho <p_c(3)$, and highly clustered.The network properties are determined by the ratio between the triangular and random mechanism of link formation , i.e., between *m* and *c*. We studied the structural properties depending on the model parameter *m* with fixed other parameters, the results are presented in Supplementary Information (Table S1, Fig. S2). Our results demonstrates that *m* is the key parameter for k-clique percolation, while the degree and clustering properties are almost independent of the value *m*. The chosen model parameters allow us to observe k-clique percolation for $k=5,6$. Degree distributions of the networks are fitted by the power law $p=Cd^{-\gamma }$ with $\gamma =2.6$, see Fig. 5a. The size of k-clique percolation cluster in dependence on the value *k* demonstrates the same behaviour as observed for free association networks (Fig. 6b) and does not depend on the network size, explaining the observed robustness of free association networks in the node-removing process by degree. Thus, the assumption of preferential attachment to an edge rather than a single word may explain clique organization in free association networks.

## Discussion: towards the explanation of Miller’s $7 \pm 2$ rule?

It is established^21^ that many phenomena concerning the processing of information by the human brain for a short period of time naturally restricted by the number of controlled items. This number is estimated by the Miller’s $7 \pm 2$ rule, which implies the restricted ability of the brain. There have been a few attempts to apply the underlying network structure to explain the Miller rule concerning the capacity limit of working memory^22,23^.

From the physiological viewpoint, three groups of mechanisms behind the limit capacity have been suggested (see,^33^ for a review). Some theories assume that representations in WM decay over time, unless decay is prevented by some form of restoration process, such as rehearsal. The second mechanism of limited resource claims that there are not enough resources for higher capacity. A resource is considered as a limited quantity that enables a cognitive function or process. According to the third mechanism, our ability to hold several representations available at the same time is limited by mutual destructive interference between these representations. As an example of this mechanism, one could have in mind the interference of frequency bands in brain activity. Indeed, a few bands are simultaneously involved in the processing of working memory. None of these mechanisms can be considered as fully satisfactory. Another network-motivated approach^34^ utilizes the mathematical result concerning the plane colouring in four colors. This idea was conjectured to be relevant to the smaller critical number of items discussed in^22,23^.

Can we gain some new insight concerning the mechanism behind Miller’s rule from our study? Let us assume that the free association tests are the specific probes of the working memory. This assumption has been discussed in the literature before ( see, for instance^35^), and it is natural because the time allowed for the performance of tests is quite restricted. Hence, let us assume that free association networks reflect the working memory and their structures code the information concerning the groups of related stimuli during the test. We conjecture that keeping the information about the linked group of k stimuli is encoded in the k-clique percolation. Hence, our finding that the k-clique percolation for the SWOW-EN and Dutch networks is possible only for $k \le 6$ can be interpreted as an example of Miller’s rule.

One concern may be that in the working memory setup, we have “percolation in time”to keep the group of stimuli as a whole for some period of time. On the other hand, in clique percolation, we have a“percolation on the network”keeping the clique intact when moving along the graph. However, to some extent, the formation of the association network can be considered as the growing network model. This viewpoint (if true) suggests the new perspective of explanation of Miller’s rule for all behavioral situations when the network description is available. One has to estimate the maximum size of a percolating clique in the particular network architecture to find the capacity of the working memory, which ensures the propagation of the linked group of items in time.

One could question why only the small-size clique percolation is possible in the human brain, although naively, we could expect that the brain would prefer the higher working memory capacity. In particular, the limit of the working memory capacity for humans is higher than of other animals^36^, and it is assumed that higher working memory corresponds to higher intellect. The answer certainly should involve some evolutionary arguments, and at least two alternative scenarios are possible. First, presumably, the network architecture admitting higher clique percolation contradicts some other vital properties of the brain encoded in connectome architecture.

Second, we can assume that the particular evolutionary rules (see,^37^ for a review) for the corresponding network dynamically bring it to the particular capacity limit somewhat in the spirit of self-organized criticality. In the previous Section, we have supported this possibility suggesting the non-conventional version of the preferential attachment procedure which indeed yields the reasonable value of $k_c=(5-6)$.

## Conclusion

In this paper, we have analyzed k-clique percolation in free association networks and semantic networks for a few languages. Some of the findings of our study which seem to be important. First, using the traditional approach we have investigated the structural network properties via the percolation theory and made a few new observations

- There is a critical value $k_c$ for the maximal size of a percolating k-clique for the SWOW-EN network and semantic networks. The larger clique with $k>k_c$ can not percolate through these networks.
- The density of the analyzed network does not allow the percolation of $k>2$ cliques if the network is considered as random. This means that our study confirms the non-randomness of the free association networks.
- Imposing the thresholds on the link weights, we investigated the role of weak and strong associations on the k-clique size and percolation. Strong associations play a key role in the k-clique percolating cluster while the weak associations provide a kind of shadow, which is necessary ingredient to support the observations made in^10^. The clear-cut dependence between averaged local weights and local connectivity was established.

Secondly, we proposed a model of generalized preferential attachment, in which the cut-off in the maximal size of percolating clique is reproduced. This phenomenon does not exist in Erdos-Renyi random networks of the same size and density, however, we can not completely exclude the possibility that the similar cut-off exists for another organization of partially random networks.

Finally, we assume that our findings provide additional information not only on the structure but also the processing on the network. Namely, the free association networks can be considered as a peculiar probe of the working memory. Therefore the critical $k_c$ for the k-clique percolation presumably can be interpreted as capacity limit of the working memory and therefore can be new, nontrivial, qualitative mechanism behind Miller’s law. This can be further checked by investigating a threshold in k-clique percolation for other cognitive networks involving short-term performances probing working memory.

It would be interesting to elaborate the possible origin of the $k_c$ actual value further. Presumably, it can be established evolutionarily^37^ as an optimal result of competition between the clique percolation related to the working memory and another properties of the connectome responsible for important cognitive properties. Another possibility demonstrated in our study is that the specific version of the preferential attachment evolution mechanism yields the critical value $k_c=(5-6)$ via a kind of self-organized criticality. It would be interesting to test our new evolutionary rule on other cognitive processes.

## Supplementary Information

Supplementary Information.

## Data Availability

All data and code are available at https://github.com/valbao/k-clique-percolation-in-semantic-networks.
