# Supplementary material for: K-clique percolation in free association networks and the possible mechanism behind the 7 ± 2 law
Source: Sci Rep. 2022 Apr 1;12:5540. doi: 10.1038/s41598-022-09499-w (PMC8975849; doi:10.1038/s41598-022-09499-w)
Supplement: Supplementary file 1 — Supplementary Information. [file 41598_2022_9499_MOESM1_ESM.pdf]

K-clique percolation in free association networks and possible mechanism behind the 7 ± 2 law.

Olga Valba[1,*] **and Alexander Gorsky**[2,3]

[1]Department of Applied Mathematics, MIEM, National Research University Higher School of Economics, Moscow,123458, Russia
[2]Kharkevich Institute for Information Transmission Problems RAS, Moscow, 127051,Russia
[3]Moscow Institute of Physics and Technology, Dolgoprudny, 141700, Russia
[*]ovalba@hse.ru

Supplementary Information

Table S1. Structural properties of simulated networks of different parameter m. The other model parameters: $n = 4000,\ c = 4,\ l = 20,\ p_0 = 0.2$.

m	Edges	Density	Transitivity	Clustering	$p_c(2)$	$p_c(3)$
1	23 924	0.0030	0.035	0.197	$2.5 \cdot 10^{-4}$	0.0111
2	31 735	0.0040	0.028	0.143	$2.5 \cdot 10^{-4}$	0.0111
3	39 330	0.0049	0.026	0.142	$2.5 \cdot 10^{-4}$	0.0111
4	47 082	0.0059	0.030	0.131	$2.5 \cdot 10^{-4}$	0.0111
5	54 547	0.0068	0.032	0.135	$2.5 \cdot 10^{-4}$	0.0111
6	61 030	0.0076	0.030	0.156	$2.5 \cdot 10^{-4}$	0.0111
7	68 800	0.0086	0.033	0.158	$2.5 \cdot 10^{-4}$	0.0111
8	76 012	0.0095	0.035	0.166	$2.5 \cdot 10^{-4}$	0.0111
9	83 376	0.0104	0.037	0.170	$2.5 \cdot 10^{-4}$	0.0111
10	90 904	0.0114	0.041	0.160	$2.5 \cdot 10^{-4}$	0.0111

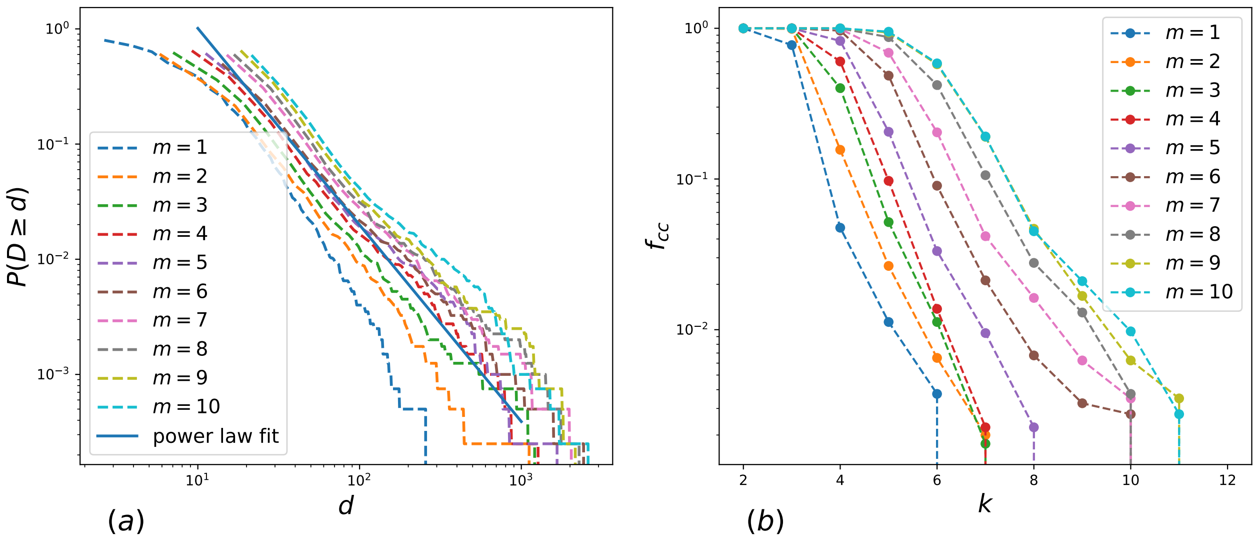

Figure S1. (a) Complementary cumulative degree distribution function for simulated networks of different parameter *m*; (b) The size of k-clique percolation cluster in dependence on the value *k* for networks of different parameter *m*.

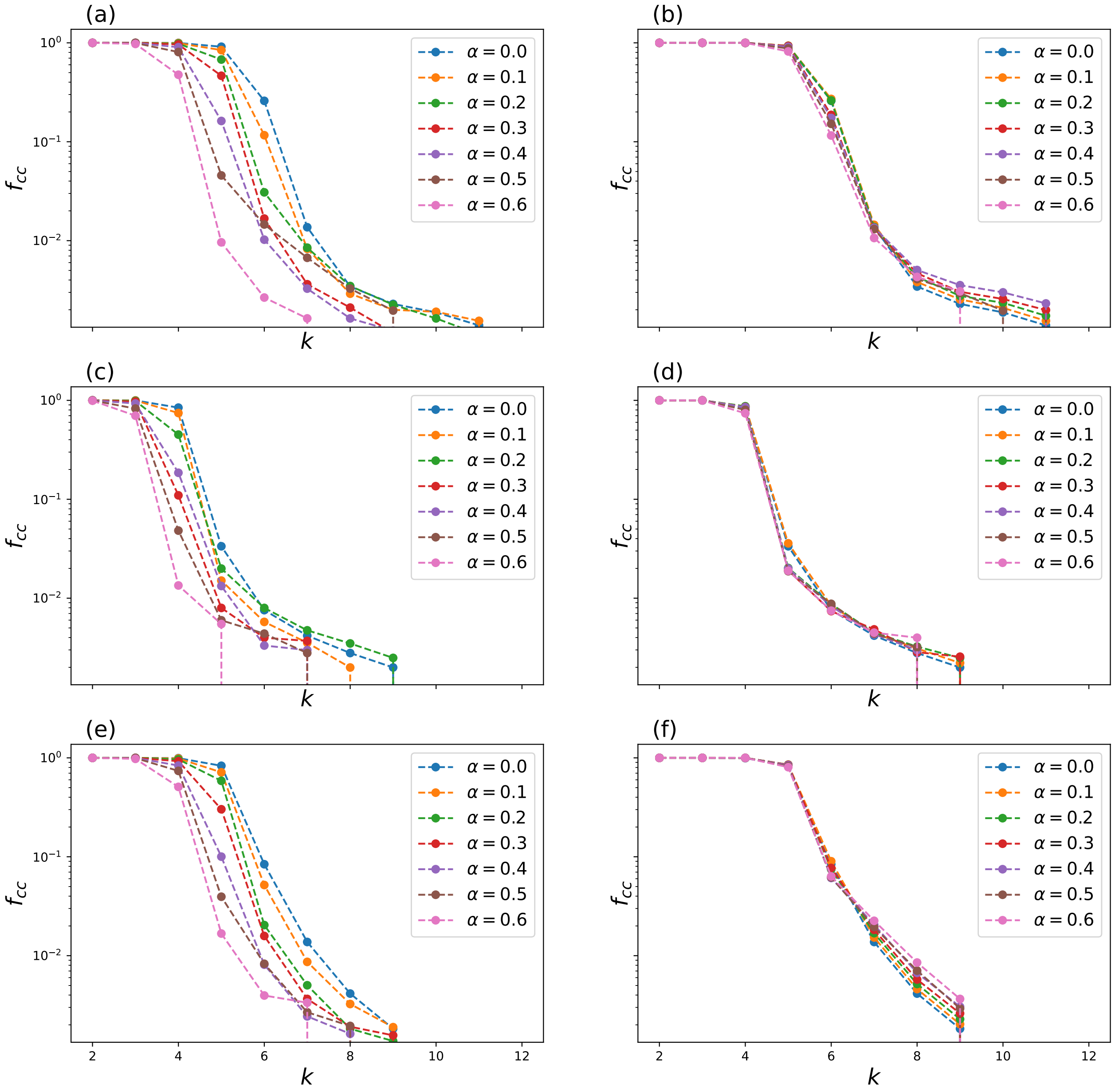

Figure S2. (a) The size of k-clique percolation cluster in dependence on the value k for different free association datasets: (a),(b) - the SWoW network; (c),(d) - the South Florida dataset; (e),(f) - the Edinburgh dataset. The right column presents numerical results for subgraphs obtained by removing randomly selected vertices from the initial network, the value α describes the fraction of the removed nodes. The left column shows the results for the subgraphs obtained by removing the vertices with the smallest degree from the initial network, the value α describes the fraction of the removed nodes.
